# Developing and Evaluating a University Recommender System

**DOI:** 10.3389/frai.2021.796268

**Published:** 2022-02-02

**Authors:** Mehdi Elahi, Alain Starke, Nabil El Ioini, Anna Alexander Lambrix, Christoph Trattner

**Affiliations:** ^1^Behavioral Data Analytics & Recommender Systems Research Group (DARS), Department of Information Science & Media Studies, University of Bergen, Bergen, Norway; ^2^Marketing and Consumer Behaviour Group, Wageningen University & Research, Wageningen, Netherlands; ^3^Software and Systems Engineering Research Group, Free University of Bolzano & Research, Bolzano, Italy

**Keywords:** recommender systems, education, offline evaluation, user study, usability, university

## Abstract

A challenge for many young adults is to find the right institution to follow higher education. Global university rankings are a commonly used, but inefficient tool, for they do not consider a person's preferences and needs. For example, some persons pursue prestige in their higher education, while others prefer proximity. This paper develops and evaluates a university recommender system, eliciting user preferences as ratings to build predictive models and to generate personalized university ranking lists. In Study 1, we performed offline evaluation on a rating dataset to determine which recommender approaches had the highest predictive value. In Study 2, we selected three algorithms to produce different university recommendation lists in our online tool, asking our users to compare and evaluate them in terms of different metrics (Accuracy, Diversity, Perceived Personalization, Satisfaction, and Novelty). We show that a SVD algorithm scores high on accuracy and perceived personalization, while a KNN algorithm scores better on novelty. We also report findings on preferred university features.

## 1. Introduction

Recommender Systems have been used in various domains to retrieve and suggest personalized content to users. Most commonly, they are used to support low-stake decisions in consumerism domains that have leisure-related goals, such as when deciding what movie to watch (Gomez-Uribe and Hunt, 2015), or what product to promote on an e-commerce website (Jannach et al., 2010). In domains where the “decision stakes” are higher, because the user decision is important or costly, the recommender approaches or algorithms should also be aimed at optimizing diversity and longer-term preferences, instead of maximizing short-term engagement only. This applies to, for instance, recommender systems that promote healthy foods or suggestions for real estate (Yuan et al., 2013; Starke and Trattner, 2021; Starke et al., 2021b).

A domain with high-stake decisions and a large potential choice set is university education. This applies to choices one can make while attending higher education, such as what college major to take and what electives to follow (Dwivedi and Roshni, 2017; Khoja and Shetty, 2017; Obeid et al., 2018), as well as to the decision of attending a university or another higher education institution. Whereas the former has been the topic of various recommender system and learning analytics approaches [cf., Hasan et al. (2016)], universities are rarely featured in personalized approaches (Rivera et al., 2018). This is arguably surprising, because a significant proportion of students attending higher education in G20 countries is not native to those countries (OECD, 2013) – even though most prospective students opt for institutions that are close to home, thus based on proximity (Simões and Soares, 2010; White and Lee, 2020). Those who would like venture further in terms of proximity, would benefit from a personalized information-filtering system, such as a recommender system, since there are over ten thousand of higher education institutions worldwide to choose from ^1^.

One's choice for higher education not only determines where one needs to move to, but also affects one's future (Kanoje et al., 2016). The recommended content should not only reflect one's current preferences, but also future prospects (Ekstrand and Willemsen, 2016), such as factors whether one wishes to focus on proximity for short-term benefits or if one seeks out prestige for long-term gains. Hence, choosing a university and enrolling into one is a vastly different experience from, for instance, watching a movie. People may watch a lot of movies during their lifetime, while experiencing universities is costly and often not feasible. One underlying reason is that people do not always have a clear idea on where to obtain helpful information, beyond the website of a specific institution and popular university rankings (Hemsley-Brown, 2012). Therefore, accessing a system that could effectively support such decision and personally help in this choice process could be as beneficial as a personal adviser would.

Current online services that assist users by ranking universities typically use explicit criteria, for example by applying decision filters (Rivera et al., 2018). Most tools are built by comparing a large number of universities, renowned colleges and schools (Hemsley-Brown, 2012). Among the most well-known resources are international university rankings, such as the World University Rankings compiled by Times Higher Education.^2^ For such ranked lists, different comparison dimensions are typically considered and weighted, and a final score is computed for each institution, for example based on a university's reputation and produced patents. Such a score could indicate how desirable a higher education institution could potentially be for a user. However, such rankings are compiled generically and are computed equally for everybody, ignoring the specific set of preferences that each person may have, which may not be reflected by such a one-size-fits-all ranking.

### 1.1. Approach

This paper addresses this problem by proposing a system that provides personalized ranking lists of universities. We go beyond current applications in the field that, in most cases, rely on search functionalities and a limited set of criteria filters, such as a university's geographical location (see CollegeBoard, 2019 for an example). We compare different algorithms that are well-known in the *Machine Learning* community, and can be used to produce a list of university recommendations. Although there have been a few studies that have attempted to build customized university rankings (Hasan et al., 2016; Bodily and Verbert, 2017; Rathore, 2017; Rivera et al., 2018; CollegeRaptor, 2019), to the best of our knowledge, none of the current world-class university rankings offer a customized ranking list that is tailored to the particular preferences and needs of the users.

We explore the effectiveness of personalized university recommendations and the users' decision-making process in more detail. This paper extends findings from our previous short paper (Elahi et al., 2020), that explored the possibilities of different Collaborative Filtering (CF) recommender algorithms, as one of the most popular types of recommender approaches. First, we compare the effectiveness of different CF-based algorithms, reporting the findings from an offline simulation study. Specifically, we measure the rating prediction accuracy of different approaches. Second, we present novel findings from our online user study, in which we have examined different aspects of university recommendation. Not only do we inquire on specific features that people (or users, for that matter) pay attention to when considering to choose a university, but we also assess which recommender approach is most suitable to model user preferences for specific universities, based on collected ratings and algorithmic evaluation. Finally, we validate the use of our recommender interface by asking users to assess the system's usability.

This work significantly extends initial findings from an offline simulation study, presenting the results of an online comprehensive evaluation methodology. To do so, we employ a wide range of validated of metrics to measuring the quality of recommendation perceived by the real users participating in the evaluation of the up-and-running university recommender system. This has been conducted using a set of beyond-accuracy metrics, including *Diversity, User Satisfaction*, and *Novelty*. To our knowledge, none of the prior works have conducted such a comprehensive experiment with similar setup in this application domain.

We examine the following research questions:

- **RQ1:** Which recommender approach has the highest predictive value when generating personalized university rankings?
- **RQ2:** How do users perceive and evaluate different university recommender approaches?
- **RQ3:** What are the most important features that users consider when choosing a university to attend?

### 1.2. Related Work

An increasing amount of data is being collected in the context of education. To make sense of this and to employ it effectively, data analytics in the context of education has become more common in the past decade. The use of so-called “learning analytics” often aims to predict a student's course performance based on interaction data (Conijn et al., 2016; De Medio et al., 2020). However, the extent to which data-driven predictions to date are robust seems to vary. For example, in the context of Learning Management Systems, using student data (e.g., interaction times, clicks) to predict course performance shows strong differences across different courses (Conijn et al., 2016). Moreover, although such techniques provide insight to the system owners and managers, they often do not help students with education-related problems, such as deciding what course to follow next.

The task of predicting a student's performance overlaps with more traditional retrieval or recommendation tasks surrounding course content. However, personal education is among the lesser-explored recommender domains (Dascalu et al., 2016; Bodily and Verbert, 2017). For one, recommender systems have been used to predict student performance as a means for intelligent tutoring systems, by assessing the difficulty of different course components (Thai-Nghe et al., 2010). The scope of educational recommender systems can vary strongly (Rivera et al., 2018), both in terms of what algorithmic approaches are used and what areas of education are covered. With regard to the former, it seems that collaborative filtering (CF) and hybrid approaches that involve a CF component are most popular (Rivera et al., 2018), arguably because Learning Management Systems (LMSs) generate a lot of interaction data from which student-related parameters can be distilled (Conijn et al., 2016; Hasan et al., 2016).

The types of areas of education covered can vary in terms of level of education, scope, and level of detail (Rivera et al., 2018). Older recommender studies in an educational context examine how individual learning tasks can be recommended. For instance, personalized recommendations to learn English and measurement models for writing ability could help systems to determine which task or assignment is suitable for which student (Engelhard, 1992; Hsu, 2008). More contemporary methods, such as through fuzzy linguistic web methods, have also been employed in the past decade to move toward personalized education approaches (Tejeda-Lorente et al., 2015). Among other approaches, recommender systems are used to suggest personalized content on e-learning platforms, as well as to generate personalized curricula for a given major or university education (Meryem et al., 2016).

Recommenders are also used to predict student performance before they enter higher education. For example, college admission recommender systems can guide higher education staff on decisions on whom to admit to their program (Ragab et al., 2012, 2014). Similar approaches have also been employed for university admission, typically using hybrid approaches (Wakil et al., 2014).

More contemporary systems aim to recommend courses or college majors (Dwivedi and Roshni, 2017; Khoja and Shetty, 2017). A recent work-in-progress proposed a method to introduce an ontology-based recommender system to help high school students to navigate college majors and to select one, along with a university (Obeid et al., 2018). However, an empirical recommender study (e.g., with crowdsourcing data) has yet to be performed, as Obeid et al. (2018) only identified the student requirements, interests, and capabilities. Nonetheless, identifying such a set of relevant features may be a good starting point to effectively perform preference elicitation.

Dwivedi and Roshni (2017) present a collaborative filtering approach to recommend elective courses to university students. This is based on a student's performance across different courses and computing the inter-item similarity between courses, which is also found in a few other proposed approaches (O'Mahony and Smyth, 2007). Other recommender approaches for courses in higher education are content- or knowledge-based, or hybrid (O'Mahony and Smyth, 2007; Khoja and Shetty, 2017). In a similar vein, such approaches also employ similar-item retrieval to generate course recommendations that are close to a course that a student is currently following. Moreover, a study that employed a knowledge-based recommender system also explored more detailed aspects of higher education, such as how to match a student to a supervisor (Samin and Azim, 2019).

Related work that suggests content at the institution level (i.e., which university to attend), particularly in an international context, is much rarer. While some recommender studies have examined university recommendation as a topic because of an interest in college major advice (Obeid et al., 2018), there is little work that examines specific characteristics of the university beyond its majors (Bodily and Verbert, 2017). Studies to date have suggested different approaches that are examined using offline evaluation. For example, Bokde et al. (2015) perform dimensionality reduction techniques (i.e., Singular Value Decomposition) in a collaborative filtering approach, based on student ratings for different criteria. The study is, however, unclear about how data was collected, which makes it hard to generalize the approach.

### 1.3. Contribution

What stands out from the corpus of related work is that few studies on university recommender systems have been performed. Moreover, those that have been reported typically rely on offline evaluation to predict user ratings and, at times, to generate a personalized list of university recommendations (Bodily and Verbert, 2017; Rivera et al., 2018). A rigorous two-step approach, in which the predictive accuracy of different recommender approaches is compared (cf. [RQ1]), after which also an online evaluation takes place (cf. [RQ2]), is much rarer.

On top of that, and in line with an earlier study (Bokde et al., 2015), we also inquire on the most important aspects for selecting a university (RQ3). This can provide insight into future university recommender interfaces, which may also involve filtering criteria, such as knowledge-based recommenders Jannach et al. (2010). Finally, to validate whether our university recommender interface is acceptable and understandable, we ask users to assess the usability of our university recommender system. Previous works on university recommender systems have mostly been evaluated offline (Rivera et al., 2018). As a result, there has been little attention for interface design and whether the presented recommendation list aligns with a user's preferences and needs. In addition to addressing this omission through a user's perceived evaluation, we will examine this through the system's perceived usability (Brooke, 1996).

## 2. Materials and Methods

This section covers how our university recommender system is set up, in terms of data collection for ratings, features, and algorithms. Moreover, we explain how we evaluated our algorithms: both for our offline and online studies.

### 2.1. Recommender System

#### 2.1.1. System Development and Procedure

**Figure 1:**
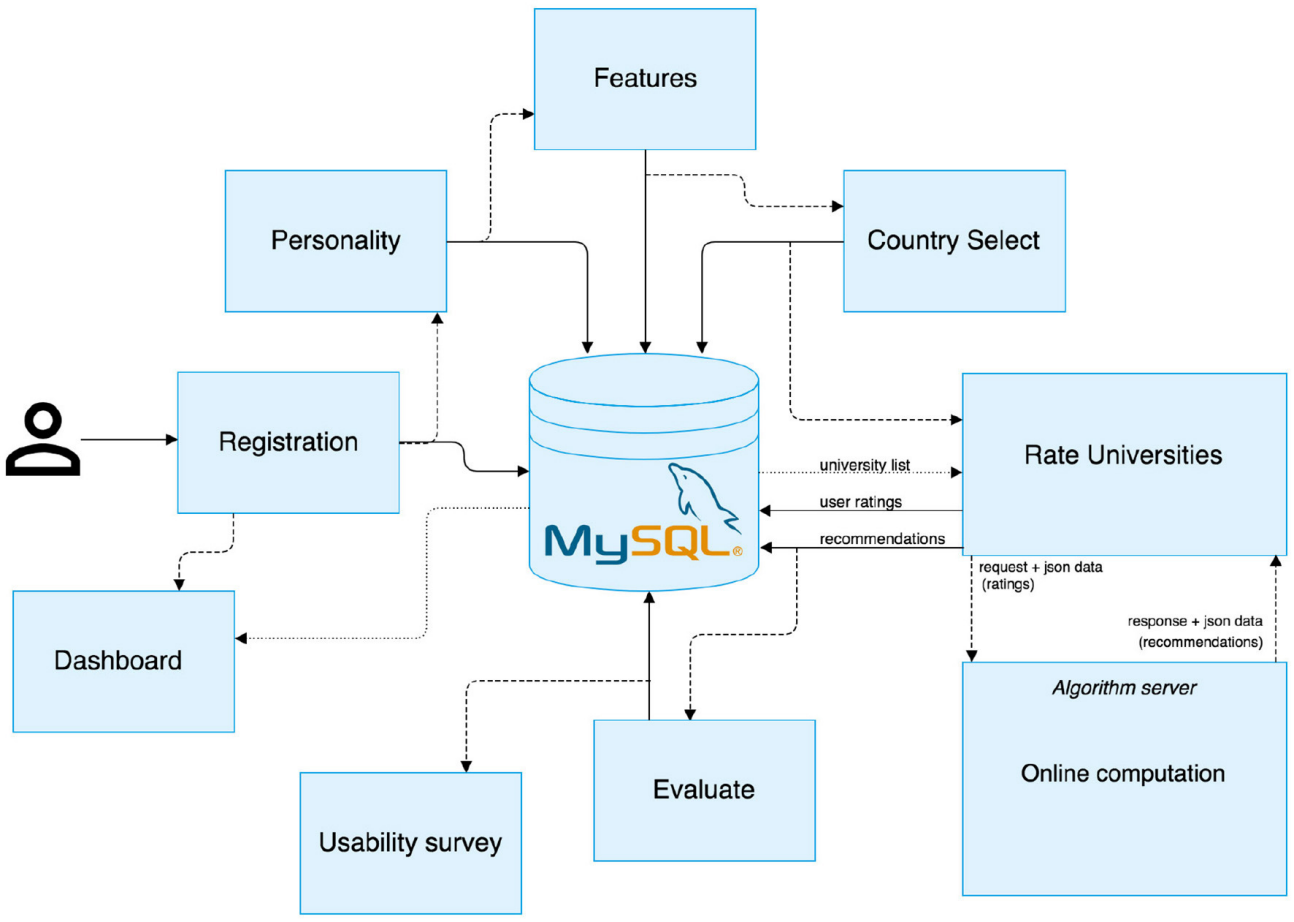
System Architecture for our University Recommender System. It depicts the flow of information in our system, as well as the different steps and features that users (depicted on the left-hand side) take when using our system.

To address our research questions, we developed a system prototype capable of interacting with users and learning their preferences for different universities. The system architecture is illustrated in Figure 1, which included several components, interconnected and operational to generate real-time recommendations of universities. When a new user entered the system (on the left of Figure 1), it initiated a *registration* process in which the system requested a user to disclose personal information. This was followed by eliciting user preferences in two different ways. First, by inquiring on what a user believed to be the most important features when choosing a university to study. Second, by asking users to rate known universities in terms of favorability. Additional information was collected in the form of personality traits, preferred university features and favorite countries. All data were passed on to a *recommender* algorithm in order to generate lists of personalized recommendations. Finally, users were requested to interact with the *evaluation* component and to assess the quality of a recommendation list.

For the development of the system, a LAMP stack was employed. This entailed the use of a APACHE server for hosting the web project's PHP files, several JS/HTML/CSS files for the front-end, and developing custom PHP scripts for the server-side processing of user data. We adopted MySQL database to store the user demographics, item descriptions, ratings, and experimental data. In order to train the recommendation engine, a separate server was utilized, based on a set of RESTful API endpoints. Finally, a customized version of WordPress content management system was developed with a specific front-end theme.

#### 2.1.2. Algorithms

We utilized multiple algorithms for our university recommendation. Most of them followed a popular recommendation approach called Collaborative Filtering (CF) (Jannach et al., 2010). Algorithms based on CF exploit ratings provided by a community of users in order to predict the unknown ratings of the items. The items with the largest predicted ratings are recommended to users (Elahi, 2014). We adopted three categories of CF algorithms in this work: Neighborhood-based, Matrix Factorization, and co-clustering.

*Neighborhood-based* approaches are a category of algorithms that calculate rating prediction using two sets of preference data: the ratings of the user for other items and the ratings of other similar users. A prominent example of such an algorithm is K-Nearest Neighbors (KNN), which would identify *k* number of neighbors that are relevant for a particular user (Jannach et al., 2010). The item's rating prediction is calculated based on how the item was rated by the users similar to the target user. The rating ${\hat{r}}_{u,i}$ for a user *u* and an item *i* was predicted as follows:


(1) $$\begin{array}{r} {\hat{r}}_{u,i}=\bar{r_{u}}+\frac{\sum_{u^{\prime }\in N_{i\left(u\right)}}sim\left(u,u^{\prime }\right)\left(r_{u^{\prime },i}-\bar{r_{u^{\prime }}}\right)}{\sum_{u^{\prime }\in N_{i\left(u\right)}}|sim\left(u,u^{\prime }\right)|} \end{array}$$

where ${\bar{r}}_{u}$ denotes the mean rating of user *u*, *sim*(*u, u*′) is a similarity metric between two users *u* and *u*′, and *N*_*i*(*u*)_ is a set of users that have similar preferences as user *u* (i.e., are part of the neighborhood set) who also rated item *i*. Similarity was computed based on a Cosine metric.

*Matrix Factorization* algorithms typically learn both of the users' and items' (latent) factors of the same size. The vectors are then computed from the user's rating. Each value of the factor vector, assigned to an item, represents how well the item describes a specific latent aspect.

User factor vectors are indicative of the specific preference of the user for each factor. A well-known example, also used in this study, is SVD (Jannach et al., 2010). The task of the factorization is to break down the matrix of ratings *R* into two smaller matrices *S* and *M*.


(2) $$\begin{array}{r} R\approx SM^{T} \end{array}$$

where *S* denotes the |*U*| × *F* matrix, and *M* denoted |*I*| × *F* matrix. *F* reflects the number of latent factors we would like to utilize. Then, predictions for the ratings are made in the following way (Funk, 2006).


(3) $$\begin{array}{r} {\hat{r}}_{u,i}=\underset{f=1..F}{\sum }s_{uf}m_{if} \end{array}$$

where *s*_*uf*_ is the degree in which the user *u* likes the latent factor *f* and the value *m*_*if*_ denotes how strong the factor *f* is in the item *i*.

*Co-clustering* is a different type of algorithmic approach that exploits groups of similar users and similar items within calculated clusters (George and Merugu, 2005; Reshef, 2015). The prediction of ${\hat{r}}_{ui}$ is computed by assigning the users and items to some clusters *C*_*u*_, *C*_*i*_ and co-cluster *C*_*ui*_:


$${\hat{r}}_{ui}={Ĉ}_{ui}+\left(\mu_{u}-{Ĉ}_{u}\right)+\left(\mu_{i}-{Ĉ}_{i}\right)$$

where Ĉ_*ui*_ is the average rating of co-cluster *C*_*ui*_, Ĉ_*u*_ is the average rating of u's cluster, and Ĉ_*i*_ is the average ratings of i's cluster, and clusters are assigned using a straightforward optimization method.

Based on these three types of recommender approaches, we evaluated seven recommendation algorithms in order to identify the best algorithm in terms of the prediction accuracy. This included two commonly used baseline algorithms: Random and SlopeOne (cf. for more details: Jannach et al., 2010, p. 41–43). Our algorithms included two types of neighborhood-based recommenders, two matrix factorization recommenders and a co-clustering approach (see above). Each of them evaluated a small dataset that contained 1,515 ratings for 551 universities (this is described in more detail in section 2.2).

For our neighborhood-based recommenders, we employed KNN Basic (i.e., “KNN1”) and KNN with Baseline (i.e., “KNN2”). The former was a simple version, while KNN with Baseline also considered the baseline rating. This was a factor that was estimated through a learning process. We set the number of neighbors for both KNN algorithms at 40. In addition, we adopted two matrix factorization (MF) recommenders: SVD and SVD++ (Koren, 2008; Koren et al., 2009). The latter was an extension of SVD as it was capable of taking into account implicit ratings (Elahi et al., 2019). The number of factors in both SVD algorithms was set to 20.

### 2.2. Dataset

To be able to recommend universities, we crawled the web to obtain data about 12,003 universities from across the world. The data included, among others, their names, country of location, and their official website URL. We used this data in the initial version of the system to collect a (small) preference rating dataset. We used a convenience sample by distributing the link of the study on the social media platforms of some of the authors. Participants were asked to provide a number of preference ratings to the universities (i.e., “Tell us what you think of these universities”) that might be familiar to them. In doing so, we obtained 1,515 ratings from 80 users, which were provided to 551 different universities. The ratings were provided in the range of [0–100], and were further utilized in our experiments to generate university recommendation lists.

In addition, we compiled a set of features that could be important to a student when choosing a university. This was based on findings from a survey administered among students from across the world in 2017 (Quacquarelli Symonds, 2017). We used these features to better understand the preferences of the users and to obtain more information about their particular interests. The list of features was as follows:

- High-quality teaching
- Low or free tuition
- Research or internship opportunities
- High employment rate among graduates
- International diversity
- Cost of food and rent in the area
- Prestigious university brand
- Party environment or extracurricular activities
- Size of the university
- Access to sports facilities and sport clubs
- Family members have attended the university.

### 2.3. Offline Evaluation Setup

We evaluated the performance of seven different recommender algorithms to generate personalized ranking lists of universities in an offline experiment (RQ1). We performed the common *k-fold cross validation* methodology, where *k* was set to 5. This randomly split the rating dataset into 5 disjoint subsets. For each fold, 1 subset would be used as a test set and the 4 other subsets as training sets, eventually averaging the predictions across the five folds.

As mentioned earlier, we evaluated five different algorithms (i.e., KNN1, KNN2, SVD, SVD++, Co-Clustering) and two baselines. The main metric used was *Root Mean Square Error (RMSE)*, which measured the prediction accuracy in terms of the deviation of predicted ratings from the actual values in the test set.

### 2.4. Online Evaluation Setup

Based on the results of the offline experiment (cf. section 3.1), we selected the best performing algorithms for further examination in our online user study. We compared Singular Value Decomposition (SVD), Basic K-Nearest Neighbor (referred to as “KNN1”) and K-Nearest Neighbor with baselines (referred to as KNN2). To this end, a demo recommender system was developed to evaluate the quality of these algorithms.

#### 2.4.1. Users

Participants were recruited through social media and at an Italian university, as a means of convenience sampling. A total of 56 participants accepted the invitation and started the experiment. Among them, only 52 selected the most important features and submitted their ratings. Eventually, 41 participants filled out the user evaluation questionnaire, while 37 participants completed the whole study. Participants provided on average 19 ratings to different universities. While all of our participants rated a minimum of 3 universities, this was somewhat skewed by one participant providing ratings to 150 different universities.

Among those that submitted the most important features, 38 identified as male, 11 as female, and 3 participants did not wish to disclose their gender. Most participants were either 18–24 (36.7%), 25–34 (38.78%), or 35–44 (22.5%) years old. Since submitting one's level of education was not compulsory, only 34 participants did so, among which the majority had obtained at least a bachelor's degree (79.4%).

#### 2.4.2. Procedure

**Figure 2:**
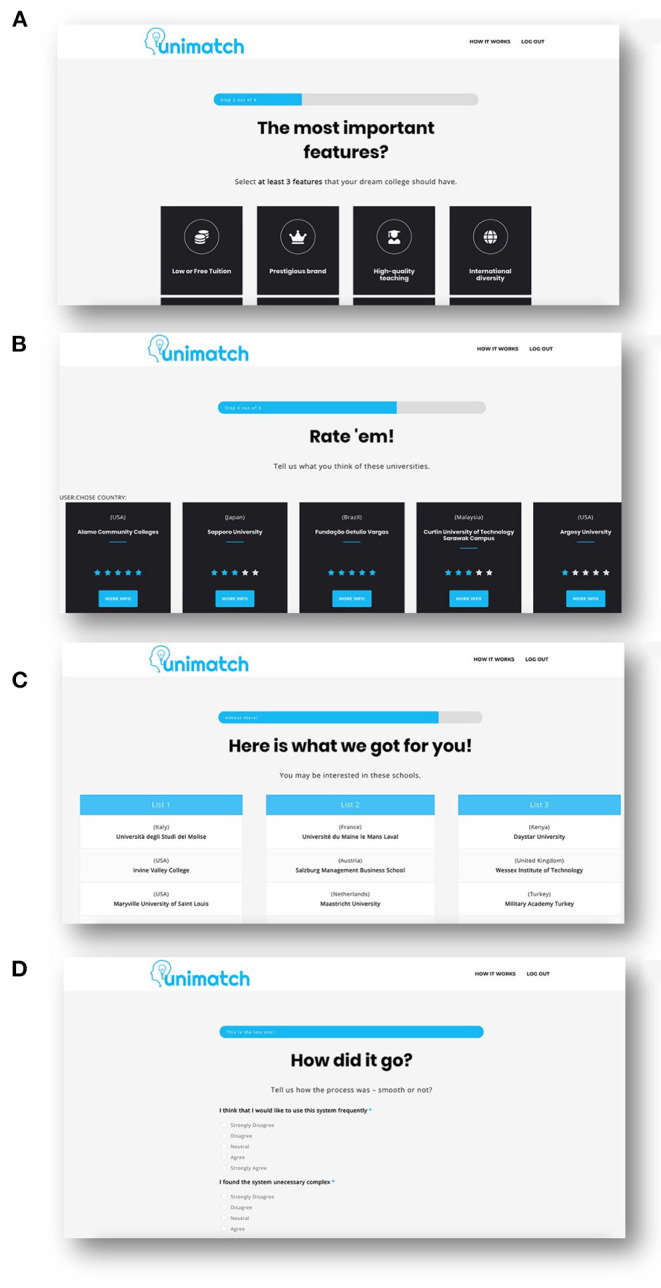
Snapshots of the system, in different stages of user interaction. In **(A)**, users had to select at least three features they found important when selecting a university. In **(B)** ,they were asked rate at least three universities, while in **(C)** they were presented three personalized recommendation lists with universities. Panel **(D)** depicts the System Usability Questionnaire. Not depicted are the demographics and user evaluation screens.

Participants were first informed about the overall goal of the experiment. After providing basic demographic information (e.g., education, age, and gender)^3^, we presented users a set of features that could possibly play an important role when making a decision on which university to choose for one's study (cf. Figure 2A).

In the main application, participants were asked to provide their preferences (through ratings) for a set of universities that might be familiar to them (see Figure 2B). They needed to focus on universities (a minimum of three) that they had either attended for education or had sufficient experience with to make a judgment. This information was used to build a user profile, which was exploited by our recommender algorithms (see Figure 2C). Participants received three lists of recommended universities, composed of five universities each, generated by the three different algorithms that had the highest accuracy in our offline evaluation. Participants were asked to carefully inspect each list and to compare them in terms of different evaluation criteria. To investigate how a user evaluated each recommendation list (RQ2), we presented users different questions in relation to the contents of the different recommendation lists and, thus, their underlying algorithms, based on different metrics (i.e., Accuracy, Diversity, Perceived Personalization, Satisfaction, and Novelty). Finally, to validate the usability of our recommender interface (see Figure 2D), we asked users to indicate to what extent they agreed with propositions from the *System Usability Survey (SUS)*.

#### 2.4.3. User Evaluation Metrics

The evaluation questionnaire consisted of fourteen questions. It was based on previous work in the movie recommender domain from Ekstrand et al. (2014), and was adapted to the university domain. Per question, users needed to select one recommendation list that would contain either the best (e.g., having the most attractive suggestions) or the worst recommendations (e.g., having the least appealing suggestions), in relation to different evaluation metrics. This setup allowed for asymmetrical user preferences, in the sense that the least chosen “best option” may not be the worst.

Different subsets of questions addressed different evaluations metrics. To address a user's evaluation of our three algorithms (i.e., SVD, KNN1, KNN2; [RQ2]), we measured the perceived *Accuracy* of a recommendation list, the perceived *Diversity* within a list, whether a user perceived that a list was personalized toward her preferences (i.e., *Understands Me*), the experienced level of *Satisfaction*, and the perceived level of *Novelty*. The list of questions was as follows, noting that some questions were formulated *positively*, while others were formulated *negatively*:

- **Accuracy:** Q1. Which list has more selections that you find appealing? [*positive*]
- **Accuracy:** Q2. Which list has more obviously bad suggestions for you? [*negative*]
- **Diversity:** Q3. Which list has more universities that are similar to each other? [*negative*]
- **Diversity:** Q4. Which list has a more varied selection of universities? [*positive*]
- **Diversity:** Q5. Which list has universities that match a wider variety of preferences? [*positive*]
- **Understands Me:** Q6. Which list better reflects your preferences in universities? [*positive*]
- **Understands Me:** Q7. Which list seems more personalized to your university ratings? [*positive*]
- **Understands Me:** Q8. Which list represents mainstream ratings instead of your own? [*negative*]
- **Satisfaction:** Q9. Which list would better help you find universities to consider? [*positive*]
- **Satisfaction:** Q10. Which list would you likely to recommend to your friends? [*positive*]
- **Novelty:** Q11. Which list has more universities you did not expect? [*positive*]
- **Novelty:** Q12. Which list has more universities that are familiar to you? [*negative*]
- **Novelty:** Q13. Which list has more pleasantly surprising universities? [*positive*]
- **Novelty:** Q14. Which list provides fewer new suggestions? [*negative*].

#### 2.4.4. System Usability

Finally, we explored the usability of our recommender interface through the System Usability Scale (SUS) (Brooke, 1996). The SUS was composed of a ten-item questionnaire with different propositions on the system's usability. The full list of items was as follows, where even-numbered propositions evaluated system aspects positively and odd-numbered propositions did so negatively:

- **P1:** I think that I would like to use this recommender system for finding the right university. [*positive*]
- **P2:** I found the recommender system unnecessarily complex. [*negative*]
- **P3:** I thought the recommender system was easy to use. [*positive*]
- **P4:** I think that I would need the support of a technical person to be able to use this recommender system. [*negative*]
- **P5:** I found the various functions in this recommender system were well integrated. [*positive*]
- **P6:** I thought there was too much inconsistency in this recommender system. [*negative*]
- **P7:** I would imagine that most people would learn to use this recommender system very quickly. [*positive*]
- **P8:** I found the recommender system very cumbersome to use. [*negative*]
- **P9:** I felt very confident using the recommender system to find my preferred university. [*positive*]
- **P10:** I needed to learn a lot of things before I can get going with the recommender system. [*negative*].

In line with Brooke (1996), we used 5-point Likert scales to measure user responses. For positive items, these responses amounted to points, ranging from -1 (Strongly Disagree) to 3 (Strongly Agree). For negative items, points yielded from responses ranged from 5 (Strongly Disagree) to 1 (Strongly Agree). After adding up the points from all items, the total score was computed by multiplying the sum with 2.5, yielding a score between 0 and 100. In this context, the average SUS score computed in a benchmark of 500 studies was 68 (Sauro, 2011).

## 3. Results

We discuss the results obtained from our studies. First, we performed an offline evaluation to investigate which algorithm had the highest predictive power in university recommendation (RQ1). Second, we performed online evaluation, by letting users in a crowdsourcing study evaluate different aspects of three recommender algorithms (RQ2). Moreover, we inquire on desirable university features (RQ3).

### 3.1. Experiment A: Offline Study

We examined which of our collaborative filtering recommender approaches had the highest predictive value when generating personalized university rankings. We evaluated each algorithm offline, by performing and computing the *Root Mean Squared Error* (RMSE) for each algorithm (Schedl et al., 2018).

The results of our offline evaluation, performed using five-fold cross-validation, are presented in Table 1. As indicated by the lowest RMSE value, the best results were obtained for the SVD algorithm, with a mean value of 23.7. In terms of predictive accuracy, the second-best performing algorithm was SVD++ (Mean RMSE = 24.1), while the third-best algorithm in terms of RMSE was KNN2 algorithm, with a mean RMSE value of 24.9. Although the random baseline produced, as expected, much worse results (RMSE = 36.5) than the other algorithms, the relatively simple SlopeOne approach performed relatively well, for it had a lower mean RMSE (26.8) than both KNN1 (RMSE = 27.7) and Co-Clustering (RMSE = 27.5).

**Table 1:** Results of the offline experiment, performed using five-fold cross-validation.

**Algorithm**	**Type**	**RMSE**			
		***Min***	***Max***	***SD***	***Mean***
SVD	-	**21.5**	**25.4**	**1.8**	**23.7**
SVD++	-	22.6	26.0	1.3	24.1
KNN1	Basic	24.9	29.2	1.7	27.7
KNN2	With baselines	23.5	25.9	1.1	24.9
Co-clustering	-	24.2	29.9	2.1	27.5
SlopeOne	-	25.7	28.3	1.1	26.8
Random	-	34.5	39.8	2.1	36.5

*SlopeOne and Random were used as baselines. Lower values of the Root Mean Square Error (RMSE) indicate that an algorithm has a higher predictive value. Denoted in bold is the best-performing algorithm in terms of RMSE*.

To proceed, we also considered how computationally demanding our algorithms were. In terms of runtime, the SVD recommender algorithm had an excellent performance, while SVD++ was the slowest among all algorithms. The latter might be due to the setup of the algorithm, which was originally proposed to work with *implicit feedbacks* (e.g., clicks) rather than *explicit feedback* (e.g., ratings). Hence, we did not consider SVD++ for our online evaluation. Instead, for the next phase, we opted for both KNN1 and KNN2. KNN1 had a short runtime combined with a somewhat worse accuracy, while KNN2 had both a comparatively good accuracy level and a decent runtime.

### 3.2. Experiment B: Online Study

#### 3.2.1. Evaluation of Recommendation Lists

We compared how users evaluated different university recommendation lists, which were generated by different algorithms. Table 2 outlines per question the percentage of instances in a which recommendation list was chosen, designated by the algorithm generating it. Some questions contributed positively to a specific metric (e.g., Q1 to Accuracy), while those denoted in italics contributed negatively to that metric (e.g., Q2).

**Table 2:** Results of paired *t*-tests on different evaluation metrics (based on Ekstrand et al., 2014), in which users were asked to choose a recommendation list in relation to specific metrics.

	**Question: Which list..**.	**% Chosen**			**Pairwise** ****t**-statistic**		
		**SVD**	**KNN1**	**KNN2**	**SVD-KNN1**	**SVD-KNN2**	**KNN1-KNN2**
	1...has more selections that you find appealing?	**51**	37	12	1.00	3.56^**^	2.36^*^
Acc	2...*has more obviously bad suggestions for you?*	**22**	**22**	56	0.00	-2.65^*^	2.65^*^
	3...*has more universities that are similar to each other?*	42	**29**	**29**	0.93	0.93	0.00
Div	4...has a more varied selection of universities?	24	32	**44**	-0.62	-1.54	-0.90
	5...has items that match a wider variety of preferences?	27	**44**	29	-1.31	-0.21	1.10
	6...better reflects your preferences in universities?	**59**	29	12	2.08^*^	4.18^***^	1.74
Und	7...seems more personalized to your preferences?	**51**	39	10	0.82	3.96^***^	2.91^**^
	8...*represents more mainstream items than your own?*	44	37	**20**	0.52	2.03^*^	1.48
	9...would better help you find universities to consider?	**51**	37	12	1.00	3.56^**^	2.36^*^
Sat	10...would you likely to recommend to friends?	**51**	34	15	1.19	3.19^**^	1.84
	11...has more universities you did not expect?	17	15	**68**	0.27	-4.21^***^	-4.61^***^
	12...*has more universities that are familiar to you?*	46	44	**10**	0.16	3.54^**^	3.33^**^
Nov	13...has more pleasantly surprising universities?	**34**	**34**	32	0.00	0.19	0.19
	14...*provides fewer new suggestions?*	44	37	**20**	0.52	2.03^*^	1.48

*Lists were generated by different algorithms (SVD, KNN1, KNN2), metrics were as follows: Accuracy (Acc), Diversity (Div), Understands Me (Und), Satisfaction (Sat), and Novelty (Nov). For positive items, the highest %'s were denoted in bold; for negative items (put in italics), the lowest %'s*.

*** *p < 0.001*,

** *p < 0.01*,

* *p < 0.05*.

To examine which algorithm had the best performance per metric, we performed pairwise *t*-tests per questionnaire item. Reported on the right-hand side of Table 2 are the *t*-statistics, while the *p*-values are indicated by asterisks in superscript. The tests were performed by creating dummy variables for each algorithm, assigning the value 1 to an algorithm if its recommendation list was chosen by a user for a specific item.

Table 2 shows that the recommender algorithms are evaluated differently across different metrics. In terms of *Accuracy* (i.e., Q1, Q2), the best results were achieved by the SVD algorithm in terms of % chosen. Although there were no significant differences between SVD (Q1: 51%) and KNN1 (37%), they both significantly outperformed the KNN2 (12%) algorithm. The difference in perceived accuracy was largest for SVD, outforming KNN2 both on question 1: *t*_(40)_ = 3.56, as well as on question 2: *t*_(40)_ = −2.65, *p* = 0.012.

In terms of *Diversity* (i.e., Q3-Q5), both KNN1 and KNN2 seemed to be chosen more frequently than SVD, and thus might be favored. For Q3, SVD (42%) was perceived as generating more similar recommendations than KNN1 and KNN2 (both 29%). However, pairwise *t*-tests indicated that these differences were not significantly different (both: *p* > 0.05). For the positively formulated questions (i.e, Q4, Q5), both KNN1 and KNN2 were selected most often, once for each item. However, similar to Q3, pairwise *t*-tests did not reveal significant differences between SVD and the KNN algorithms.

Table 2 further suggests that the SVD algorithm was evaluated as generating the most favorable recommendation lists for our *Understands Me* and *Satisfaction* metrics. SVD was selected significantly more often (59%) than KNN1 (29%) for Q6: *t*_(40)_ = 2.08, *p* < 0.05, as well as than KNN2 (12%): *t*_(40)_ = 4.18, *p* < 0.001. SVD also performed significantly better than KNN2 on Q7 (*p* < 0.001), as did KNN1 (*p* < 0.01), both suggesting that users felt better understood by SVD than the KNN algorithms, particularly KNN2. Furthermore, we found that the recommendation lists generated by the SVD algorithm (51%) led to higher levels of satisfaction than those produced by KNN2 (12–15%); both for Q9: *t*_(40)_ = 3.56, *p* = 0.001, as well as for Q10: *t*_(40)_ = 3.19, *p* = 0.003. In contrast, SVD did not significantly outperform KNN1 on these questions (i.e., 34–37%), while a pairwise *t*-test indicated that KNN1 was selected more often KNN2 for Q9 (*p* = 0.023). In contrast with the favorable findings for SVD, we also observed that it was selected most often for our negatively formulated item Q8 (44%), suggesting that it produced more mainstream items than the KNN2 algorithm (20%): *t*_(40)_ = 2.03, *p* = 0.049. It was suggested that our users did not seem to interpret “mainstream items” necessarily as a negative aspect, while similar validation problems were also observed by Ekstrand et al. (2014).

Finally, for our *Novelty* metric, KNN2 was the best performing algorithm for the majority of the items (Q11, Q12, Q14). As shown by pairwise *t*-tests (cf. Table 2), most users (i.e., 68%) indicated that the KNN2 algorithm recommended more unexpected universities (Q11) than SVD and KNN1: *t*_(40)_ < −4.2, *p* < 0.001. Similar effects were observed for Q12 and Q14, as KNN2 generated significantly fewer recommendation lists that consisted of familiar universities than SVD and KNN1 [*t*_(40)_ > 3.30, *p* < 0.01], as well as was selected the least often for our item on “provides fewer new suggestions” (20%; significantly less than SVD). Finally, in contrast, Table 2 did not outline any significant differences between all three algorithms for Q13, as user choices were distributed almost equally between them. This could be attributed to the combination of “pleasantly” and “surprising” compared to use of the word “surprise” only in other questionnaire items.

#### 3.2.2. University Features

We further examined which university features were found by users to be the most important when choosing a university. As indicated in the method section, the features were obtained from a marketing agency that administered a questionnaire among students worldwide (Quacquarelli Symonds, 2017), while users could select multiple features to be important. On average, female users selected 4.9 features, while male users selected 3.9 features.

Table 3 presents an overview of the selected features, in descending order of how often they were selected, as well as divided across self-identified genders. Both for males and females, the most important features were *High-quality teaching* (80.8% across all users) and *Low or free tuition* (55.8%). Overall, *Research or internship opportunities* (51.9%) was found to the third most important feature, but was relatively speaking more important to male users than females, as *High graduate employment rate* was selected by 72.7% of female users. Hence, we observed varying priorities, also for lower-ranked features. For example, Table 3 describes that 36.4% of female users valued *Access to sports facilities and sports clubs*, while only 15.8% of male users did so. Interestingly, almost no participants considered the feature *Family members have gone to that university* in their university decision-making, deeming it to be least important feature.

**Table 3:** Self-reported features that are most important to users when choosing a university to attend for study, distributed across females and males.

**Feature**	**All (*n* = 52)**	**Female (*n* = 11)**	**Male (*n* = 38)**
	**[%]**	**[%]**	**[%]**
1. *High-quality teaching*	80.8	90.9	81.6
2. *Low or free tuition*	55.8	72.7	50.0
3. *Research or internship opportunities*	51.9	63.6	50.0
4. *High graduate employment rate*	42.3	72.7	36.8
5. *International diversity*	36.5	54.5	31.6
6. *Cost of food and rent in the area*	36.4	29	31.6
7. *Prestigious brand*	32.7	27.3	34.2
8. *Party environment or extracurricular activities*	26.9	27.3	28.9
9. *Size of the university*	23.1	9.1	23.7
10. *Access to sports facilities and sport clubs*	19.2	36.4	15.8
11. *Family members have gone to that university*	1.9	0	2.6

*Users could select multiple features. Three users did not wish to disclose their gender and are only considered in the “All” column*.

#### 3.2.3. Usability

Finally, we validated the use of our recommender interface by administering a questionnaire on the System Usability Scale (SUS) (Brooke, 1996). The results of our ten-item questionnaire with 5-point Likert scale are outlined in Table 4, noting that the average SUS score computed in a benchmark of 500 studies was 68 (Sauro, 2011).

**Table 4:** Frequencies of user responses to questionnaire items (i.e., propositions, such as “P1”) from the System Usability Scale (SUS) (Brooke, 1996).

**Items**	**Likert Scale Responses**					**Score**
	**S. disagree**	**Disagree**	**Neutral**	**Agree**	**S. agree**	
P01 Positive	3	4	9	16	5	1.43
P02 Negative	10	16	10	1	0	3.95
P03 Positive	1	1	4	16	15	2.16
P04 Negative	21	10	4	2	0	4.35
P05 Positive	1	2	11	19	4	1.62
P06 Negative	7	20	7	3	0	3.84
P07 Positive	0	2	2	18	15	2.24
P08 Negative	12	18	7	0	0	4.14
P09 Positive	0	2	8	19	8	1.89
P10 Negative	18	12	4	2	1	4.19

*Items were measured using 5-point Likert scales. Items measured whether a usability aspect was evaluated either positively or negatively. The SUS score, which was the sum of the denoted scores multiplied with 2.5, was 74.5 out of 100, which suggested that our system had a good usability (Brooke, 1996)*.

Table 4 describes that most participants evaluated the usability of the demo university recommender system higher than the benchmark score. While the actual scores given by users ranged from 42.5 (lowest) to 100 (highest), the mean score was 74.5, which indicated that our university recommender system had a good usability (Brooke, 1996). This can be observed in Table 4, for most users responded with “Agree” or “Strongly Agree” to positive items, while “Disagree” was most common for negative items. This all indicated that most of the participants evaluated the system's usability positively.

Users could also fill out an open-ended text box at the end of the study to leave comments. We reviewed the comments of users who evaluated the system with a score below 51, which entailed 3 out of 37 users. Only a single user provided a comment in which she expressed concerns regarding the lack of clarity on which criteria she was supposed to rate the universities.

## 4. Conclusion

Overall, the presented results in the offline and online studies seemed promising. They reflected the potential effectiveness of the proposed university recommender system, opening up the possibility to generate personalized ranking lists of universities in the near future. It seemed that the SVD algorithm was most appropriate to do so, based on accuracy, perceived personalization, and satisfaction. These results illustrated the potential of such a system and its importance in supporting individuals who are searching for the best universities for their future studies, which is a high-stake decision and, therefore, unlike most other recommender systems.

## 5. Discussion

This paper has addressed a challenging recommendation problem in the domain of education, specifically about where one should follow their university education. The existence of a large number of educational institutions has exacerbated the task of choosing where to study. We have described the design and development of a demo system that can provide a personalized ranking list of universities. To that end, we have compared different recommender approaches and algorithms, both in an offline and online evaluation context.

In particular, this work has attempted to address the following research questions:

- **RQ1:** Which recommender approach has the highest predictive value when generating personalized university rankings?
- **RQ2:** How do users perceive and evaluate different university recommender approaches?
- **RQ3:** What are the most important features that users consider when choosing a university to attend?

In relation to RQ1, we have found that the SVD and “k-Nearest Neighbor with baselines” approaches performed best in terms of predictive accuracy. The main metric we have used is RMSE, which indicates that we have focused on predicting the ratings in a dataset using training and test split. Although the list of approaches considered is by no means exhaustive (i.e., it only comprises collaborative filtering recommender approaches), we have shown which algorithms among a set of common approaches performed best. In addition, we have also considered runtime as a pragmatic factor on deciding which algorithms to consider for our online evaluation. This has led us to select SVD, KNN basic (i.e., “KNN1”), and KNN with baselines (i.e., “KNN2”) for the online evaluation.

With regard to RQ2 and RQ3, our findings illustrate that recommending a university to a prospective user (e.g., a student) out of the existing large number of universities is a complex problem. This is arguably due to the various dimensions and features that are involved when a user makes this choice, as well as the complexity and multi-facetedness of the recommendation items in question. Furthermore, we have noticed that preferences for a particular recommendation algorithm may vary for different users considering different features when choosing the university to study, as well as that the performance of different recommender algorithm depends on the evaluation metric in question.

Overall, we have found our SVD approach to outperform the KNN approaches on accuracy and “fit-related” metrics. Although not all differences have been found to be statistically significant, SVD outperformed the KNN approaches on perceived accuracy, perceived personalization, and satisfaction. In contrast, it is suggested that KNN2 (i.e., the KNN approach with baselines) outperforms SVD in terms of more exploratory aspects, such as diversity and novelty, although the differences for diversity have not been found to be statistically significant. Based on the findings in, among others, Table 2, it is suggested that SVD may be more suitable for users who already have a better understanding of what they are looking for and who wish to reduce the set size of potential universities. In contrast, the KNN approaches seem to be more suitable to users who are still exploring the space of possible universities.

A few of the results in the evaluation questionnaire are found to be somewhat inconsistent within a single metric. For one, it has not become clear whether KNN1 or KNN2 performs better on diversity, although the differences are rather small in the first place. Another peculiar outcome is that the perceived personalization (i.e., “Understands Me”) for SVD is found to be highest for the two positively formulated items, but also highest for the negatively formulated item. However, it seems that the wording of Q8 in Table 2, specifically mentioning “mainstream items,” made it also relate to other aspects, arguably a lack of diversity. Such an explanation, that the item does not correctly measure the “Understands me” metric, is consistent with the findings from Ekstrand et al. (2014), where the item is also found to be less related to perceived personalization than the other items used. We argue that a similar problem is also observed for Q13, which seems to measure serendipity (“pleasant” and “surprising,” Ge et al., 2010) instead of novelty; this item also seemed to not fully measure novelty in a previous study (Ekstrand et al., 2014).

We have further obtained evidence that some features seem to play a more important role in university selection than others (RQ3). In the overall top-4, we have found study and career-related features, such as *high-quality teaching, research of internship opportunities*, and *high graduate employment rate*, but also a feature that is more related to feasibility (*low or free tuition*). Features that are more related to contextual factors (i.e., cost in the area, facilities, party environment) are found to be less important, nor have we found that prestige and familiarity are particularly important. With regard to the latter, almost none of our participants have indicated that it is important that family members have attended a particular university, even though this has been part of the widespread questionnaire among university students (Quacquarelli Symonds, 2017).

### 5.1. Limitations

We would like to point out a few limitations to our online study. First, the use of a convenience sample might have reduced the quality of the collected data. We have sent out a web link to our potential participants, through the social network and personal channels of part of the research team. As a result, this has reduced the control we could exert over the type of participants that enrolled in the study and might have skewed the distribution of demographics in the sample toward men and people who have attended a university education. Moreover, this might have affected the extent to which users were actually interested in selecting a university and whether anything was “at stake” for them. However, we argue that the large proportion of participants that has attended a university education in our Study 2 sample, increases the likelihood that the task has been relevant to them. It could be argued that this makes their judgments more valid than participants who do not have such experience or “vested interests.” Nonetheless, we wholeheartedly recommend a user study to be conducted among a sample of participants that still needs to select a university education, such as high school students (16–18 year olds), who have a clear incentive to take this task seriously. We would envision a longitudinal study design that assesses whether students that followed the recommendations of a personalized ranking system have a lower drop-out rate than students who have obtained their information in different ways.

A more general limitation of online evaluation is that one cannot account for possible failures of a participant's internet connection. However, this does not seem to have played a large role in the collected data, as the dropout during the study was acceptable.

A potential lack of recognition among the recommended universities has made it possibly harder for users to assess the presented items. We have attempted to mitigate this by providing “More Info” buttons alongside each university, which would take the user to the website of that institution. However, we have not monitored whether these have been used extensively. Nonetheless, we argue that a lack of recognition among users will be a generic challenge in developing any type of recommender system in the university domain. We feel that this can be mitigated by, for instance, effective interface design that also focuses on helping users to explore new universities and obtain new information. With respect to that, we have found that the overall usability of our system is good, but this questionnaire has not inquired on specific interface aspects and user goals (e.g., exploration vs. exploitation).

### 5.2. Future Work

Since our work concerns a new application domain, that of personalized ranking and recommendation of universities, there is still more research that needs to conducted. First and foremost, it is still unclear to what extent receiving a personalized university ranking and auxiliary advice has benefits over offering a non-personalized ranking that can be found on various websites, such as The Times Higher Education ranking. Although our novel system serves as a proof-of-concept, in the sense that it can both elicit user preferences for universities through ratings and construct a personalized university ranking list, we have only compared different algorithmic baselines. Nonetheless, we have observed that recommender approaches outperform a random baseline, but it is likely that a popularity baseline will perform somewhat better.

As future work, we plan to conduct more experiments with larger datasets in terms of the number of ratings provided to universities. We will also conduct user studies to consolidate algorithms that can learn from other sources of information, such as the social media profiles of users. We will also redesign the user interface and improve the interaction model by taking advantages of novel design elements (Cremonesi et al., 2017).

Another future direction can be the development of recommender approaches that consider future goals, which has been explored in some recommender domains (Ekstrand and Willemsen, 2016; Starke et al., 2021a). The algorithms used in the current studies are not much different from those used in traditional domains and are therefore likely to optimize for short-term engagement (Jannach et al., 2010). This contrasts with the conception that one's university education benefits one in the longer-term and has a big impact one's future career and life (Rivera et al., 2018). This is, however, a broader problem in recommender system research (Ekstrand and Willemsen, 2016), also in other high-stake domains, such as healthy eating (Elsweiler, 2019).

Finally, we are planning to incorporate personality information, provided by the users, in the prediction model. This may enable the algorithms to generate ranking lists that suit the personality characteristics of the users. In the context of educational recommender systems, the use of personality traits is—to the best of our knowledge—a new approach. The types of approaches used in previous studies are generally memory-based or use a combination of collaborative filtering and content-based recommendation (Bodily and Verbert, 2017; Rivera et al., 2018). Nonetheless, one's personality has been considered as an important trait in studies in the general education domain and is, therefore, possibly a feasible predictor in determining future preferences. For example, although the existence of learning styles is currently being questioned in many studies (Riener and Willingham, 2010), it has been used in the past to tailor educational content to a person's learning style (Felder and Silverman, 1988; Rovai, 2003). The same principle, exploiting the relation between personality traits and learning styles, has also been exploited in a learning analytics and Learning Management Systems (Halawa et al., 2015).

Based on the feature importance that we have collected as part of RQ3 and existing metadata of universities, it might also be possible to pursue knowledge-based approaches, for these are more uncommon in past recommender systems in the education domain (Bodily and Verbert, 2017; Rivera et al., 2018). We argue that this and the aforementioned personality-based approaches, could be appropriate for the university domain and can overcome possible cold-start problems. In our view, one-size-fits-all rankings can easily be replaced by recommender systems, for they more effective and are more efficient in the long-run.

## Data Availability Statement

The raw data supporting the conclusions of this article will be made available by the authors, without undue reservation.

## Ethics Statement

Ethical review and approval was not required for the study on human participants in accordance with the local legislation and institutional requirements. The patients/participants provided their written informed consent to participate in this study.

## Author Contributions

ME was responsible for generating the initial idea, the write-up overall, particularly to the method and the discussion sections, and also helped to collect data. AS contributed to the Introduction, Related Work, and Results sections and performed the statistical evaluation of the online study. NE and CT supervised the process and helped to finalize the manuscript, while NE also generated the idea. AL helped to collect the data and performed the offline evaluation. All authors contributed to the article and approved the submitted version.

## Funding

This work was supported by industry partners and the Research Council of Norway with funding to MediaFutures: Research Centre for Responsible Media Technology and Innovation, through the Centres for Research-based Innovation scheme, project number 309339.

## Conflict of Interest

The authors declare that the research was conducted in the absence of any commercial or financial relationships that could be construed as a potential conflict of interest.

## Publisher's Note

All claims expressed in this article are solely those of the authors and do not necessarily represent those of their affiliated organizations, or those of the publisher, the editors and the reviewers. Any product that may be evaluated in this article, or claim that may be made by its manufacturer, is not guaranteed or endorsed by the publisher.

## Footnotes

^1^Please check *Webmetrics* which already indexed tens of thousands of universities: http://www.webometrics.info

^2^ https://www.timeshighereducation.com/world-university-rankings

^3^We also inquired on a user's personality characteristics using scales from the Big Five Inventory, but we did not consider these for analysis in the current study.
